# Endoglycan Regulates Purkinje Cell Migration by Balancing Cell-Cell Adhesion

**DOI:** 10.3389/fnins.2022.894962

**Published:** 2022-06-20

**Authors:** Thomas Baeriswyl, Martina Schaettin, Simone Leoni, Alexandre Dumoulin, Esther T. Stoeckli

**Affiliations:** Department of Molecular Life Sciences and Neuroscience Center Zurich, University of Zurich, Zurich, Switzerland

**Keywords:** cerebellum development, neural circuit development, chicken embryo, sialomucins, cell adhesion, Purkinje cells

## Abstract

The importance of cell adhesion molecules for the development of the nervous system has been recognized many decades ago. Functional *in vitro* and *in vivo* studies demonstrated a role of cell adhesion molecules in cell migration, axon growth and guidance, as well as synaptogenesis. Clearly, cell adhesion molecules have to be more than static glue making cells stick together. During axon guidance, cell adhesion molecules have been shown to act as pathway selectors but also as a means to prevent axons going astray by bundling or fasciculating axons. We identified Endoglycan as a negative regulator of cell-cell adhesion during commissural axon guidance across the midline. The presence of Endoglycan allowed commissural growth cones to smoothly navigate the floor-plate area. In the absence of Endoglycan, axons failed to exit the floor plate and turn rostrally. These observations are in line with the idea of Endoglycan acting as a lubricant, as its presence was important, but it did not matter whether Endoglycan was provided by the growth cone or the floor-plate cells. Here, we expand on these observations by demonstrating a role of Endoglycan during cell migration. In the developing cerebellum, Endoglycan was expressed by Purkinje cells during their migration from the ventricular zone to the periphery. In the absence of Endoglycan, Purkinje cells failed to migrate and, as a consequence, cerebellar morphology was strongly affected. Cerebellar folds failed to form and grow, consistent with earlier observations on a role of Purkinje cells as Shh deliverers to trigger granule cell proliferation.

## Introduction

In the cerebellum, like in other parts of the nervous system, cells proliferate in specific zones, from where they migrate to their final destinations. Once they have reached their final position, neurons start to extend processes, axons and dendrites, to connect to their targets and form synapses, and thus, establish neural circuits. The cerebellum has two proliferative zones, the ventricular zone, where most cell types have their origin, and the external granular cell layer, where the granule cells are born (Butts et al., 2014; Leto et al., 2016; Beckinghausen and Sillitoe, 2019). Cells migrate either radially or tangentially from their place of birth to their final destination. For radial migration, they are supported by radial glia fibers. Migrating cells interact with the processes of radial glia cells to find their way toward the periphery, away from the ventricular zone (Rahimi-Balaei et al., 2018). For tangential migration, the signals that guide migrating cells have not been identified. In analogy to axon guidance, where the growth cone rather than the cell body needs to navigate through the three-dimensional tissue, the idea is that specific guidance cues, recognized by surface receptors on the migrating cell, will allow for navigation to the final position. An early hypothesis suggested that proteases would allow a growth cone to ease its way through the pre-existing tissue held together by cell adhesion molecules (Krystosek and Seeds, 1981a; Seeds et al., 1997). Therefore, proteolytic support for cells migrating through the tissue might also be necessary. Indeed, granule cells *in vitro* were shown to exhibit protease activity (Krystosek and Seeds, 1981b). Although the image of a larva eating its way through food may not be an appropriate analogy to a growth cone navigating or a cell migrating through neural tissue, there is still a need to regulate adhesive strength between the migrating cell and the environment to allow advance.

The neural cell adhesion molecule NCAM was the first cell-cell adhesion molecule to be identified in the nervous system (Edelman, 1983). Interestingly, it had a regulatory mechanism for adhesive strength built in. The post-translational modification with poly-sialic acid added to some NCAM isoforms was found to lower not only the adhesion between NCAM molecules but also to interfere with the interactions of other cell adhesion molecules of the immunoglobulin superfamily, such as NgCAM (Rutishauser and Landmesser, 1996; Kiss and Rougon, 1997).

Recently, we identified a related mechanism for the regulation of adhesive strength between growth cones and their intermediate target during axon guidance (Baeriswyl et al., 2021). Commissural growth cones and the floor plate, their intermediate target, both express Endoglycan, a molecule that is heavily glycosylated in its extracellular part. Unlike NCAM, Endoglycan is not suggested to act as a receptor or ligand, but rather as lubricant, allowing growth cones to advance through the three-dimensional tissue. This may be different in the hematopoietic system, where Endoglycan was suggested to be a ligand for L-Selectin (Fieger et al., 2003).

Endoglycan, also known as Podocalyxin-like-2, belongs to the CD34 family of sialomucins, which also comprises CD34 and Podocalyxin (Sassetti et al., 2000; Furness and McNagny, 2006; Nielsen and McNagny, 2008). Sialomucins are single-pass transmembrane proteins with a bulky extracellular domain that is negatively charged due to its extensive N- and O-glycosylation.

Our studies demonstrated that during axon guidance, Endoglycan could either be provided by the growth cone or by the floor plate, but it was necessary to lower the adhesion between the migrating growth cone and the floor-plate cells during midline crossing (Baeriswyl et al., 2021). In the absence of Endoglycan, the excessive adhesive strength prevented the smooth passage of growth cones. As a result, floor-plate cells were “torn out” of the floor plate and dislocated into the commissure beneath the floor plate. In an *ex vivo* preparation for live imaging of commissural axon navigation (Dumoulin et al., 2021), the growth speed was decreased due to too much adhesion, most likely contributing to the pathfinding errors observed at the floor-plate exit site (Baeriswyl et al., 2021). Too much Endoglycan also perturbed the correct navigation of the floor-plate area by commissural axons, in agreement with the idea that adhesive strength needs to be set within a certain range.

Interestingly, a recent study not only confirmed the role of Endoglycan as an anti-adhesion molecule, but also went one step further in showing that the level of Endoglycan expression on the cell surface was under the control of ADAM10, an α-secretase capable of shedding proteins from the cell surface (Hsia et al., 2021).

Here, we extend our previous studies and demonstrate that Endoglycan is not only acting as a negative regulator of adhesion for navigating growth cones at their intermediate target but that Endoglycan also supports cell migration. Removing Endoglycan from Purkinje cells prevented their radial migration during cerebellum development. Because Purkinje cells did not reach their final position, they failed to provide Shh, the proliferation-inducing signal, to granule cell precursors. As a consequence the number of granule cells was reduced, which in turn reduced the size of the cerebellar lobes (De Luca et al., 2016).

## Materials and Methods

### Preparation of Digoxygenin-Labeled RNA Probes and *in situ* Hybridization

For *in vitro* transcription 1 μg of the linearized and purified plasmid encoding Endoglycan (EndoORF: 1028-1546pb, Endo3’UTR: 3150-3743bp, and 5070-5754bp; numbers are derived from the human sequence), were used to prepare DIG-(digoxygenin)-labeled *in situ* probes as described earlier (Mauti et al., 2006). The same fragments were used to prepare dsRNA (Pekarik et al., 2003).

### *Ex ovo* RNAi

All experiments including animals were carried out according to the guidelines and regulations of the Cantonal authorities (Veterinäramt des Kanton Zürich). To analyze the *in vivo* function of Endoglycan in the developing cerebellum *ex ovo* cultures of chicken embryos were prepared (Baeriswyl and Stoeckli, 2008). Injections and electroporations were performed at E8 (HH34). To have direct access to the embryo a small hole of 3–4 mm diameter was cut into the extraembryonic membranes above the eye. For positioning and stabilization of the head during injection and subsequent electroporation, we used a hook prepared from a spatula. Approximately 1 μl of the nucleic acid mixture, consisting of a plasmid encoding EGFP under the control of the β-actin promoter (100 ng/μl), dsRNA derived from the ORF of *Endoglycan* (500 ng/μl), and 0.04% (vol/vol) Trypan Blue (Invitrogen) dissolved in sterile PBS, were injected into the cerebellum using a borosilicate glass capillary with a tip diameter of 5 μm (World Precision Instruments). Before electroporation, a few drops of sterile PBS were added to the embryo. For the electroporation, a platelet electrode of 7 mm diameter (Tweezertrodes Model #520, BTX Instrument Division, Harvard Apparatus) was placed parallel to the head of the embryo. Six pulses of 40 V and 99 ms duration were applied using a square wave electroporator (ECM830, BTX).

Efficiency and specificity of Endoglycan downregulation with the long dsRNA derived from *Endoglycan*, which was used here, was verified and quantified in detail in our previously published study on the role of Endoglycan in commissural axon guidance in the spinal cord (Figure 2 and Supplementary Figure 3 in Baeriswyl et al., 2021). In short, we used two different non-overlapping sequences from the 3’-UTR and one sequence from the ORF of *Endoglycan* to produce long dsRNA. The phenotypes resulting from electroporation of the three different dsRNAs did not differ. Silencing other CD34 family members did not interfere with commissural axon guidance. The *Endoglycan* mRNA levels in the electroporated area were markedly reduced, reaching about 80% of the theoretical maximum given that about 50% of the cells were transfected.

### Tissue Preparation and Analysis

The embryos were sacrificed for the analysis of the cerebellum 4 days after electroporation. The whole brain was removed and analyzed for EGFP expression using a fluorescence stereomicroscope (Olympus SZX12). The brain tissue was fixed for 2 h at room temperature in 4% PFA in PBS. After fixation, the brain tissue was rinsed in PBS and transferred to 25% sucrose in 0.1M sodium phosphate buffer, pH 7.4, for cryoprotection. In this study, 30 μm-thick sagittal cryostat sections were used for analysis. For the preparation of cryostat sections, the brains were embedded with O.C.T Tissue-Tek (Sakura) in Peel-a-Way disposable embedding molds (Polysciences), frozen in isopentane on dry ice and cut on a cryocut (CM1850, Leica Microsystems). The sections were collected on SuperFrostPlus microscope slides (Menzel-Glaeser).

### Immunohistochemistry

Cryostat sections were rinsed in PBS at 37°C for 3 min followed by 3 min in cold water. Subsequently the sections were incubated in 20 mM lysine in 0.1 M sodium phosphate (pH 7.4) for 30 min at room temperature before being rinsed in PBS three times for 10 min. The tissue was permeabilized with 0.1% Triton in PBS for 30 min at room temperature and then washed again three times with PBS for 10 min. To prevent unspecific binding of the antibody the tissue was blocked with 10% fetal calf serum (FCS) in PBS for 1 h. Goat anti-GFP (1:400; Rockland), rabbit anti-Calbindin D-28K (CB38a; Swant), mouse anti-Pax6 (2 μg/ml; Developmental Studies Hybridoma Bank) were dissolved in 10% FCS/PBS and incubated overnight at 4°C. After three washes in PBS, 10% FCS in PBS was applied again for 1 h, followed by the incubation with donkey anti-rabbit IgG-Cy3 (1:250; Molecular Probes), donkey anti-goat IgG-Alexa488 (1:250; Molecular Probes/Invitrogen) and goat anti-mouse IgG-Cy3 (1:250; Jackson ImmunoResearch) dissolved in 10% FCS in PBS for 90 min at room temperature. The tissue was rinsed 5 times in PBS for 12 min and then mounted in Celvol (Celanese). The staining of cryostat sections was analyzed with an upright microscope equipped with fluorescence optics (Olympus BX51).

### Analysis of Cell Proliferation and Cell Death

To assess cell proliferation in the developing cerebellum, we used BrdU incorporation. Embryos were injected and electroporated at HH34 with dsRNA derived from *Endoglycan* and the *EGFP* plasmid or with the *EGFP* plasmid alone. After 1 (HH35) or 4 days (HH38) 200 μl 50 mM BrdU in H_2_O were pipetted onto the chorioallantois. After 3 h the embryos were sacrificed, the brains were dissected and prepared for cryostat sections as described above. For visualization of the incorporated BrdU, the sections were incubated in 50% formamide in 2xSSC for 1–2 h at 65°C, rinsed twice in 2xSSC for 15 min followed by incubation in 2 N HCl for 30 min at 37°C. Sections were rinsed in 0.1 M borate buffer (pH 8.5) for 10 min at room temperature, followed by PBS (six changes). BrdU was detected with mouse anti-BrdU (Sigma; 1:200) using the protocol detailed above. Sections were counterstained with DAPI (5 μg/ml in PBS) for 20 min at room temperature.

Apoptosis was analyzed as described previously (Baeriswyl and Stoeckli, 2008). In brief, to detect apoptotic cells, the ApoAlert DNA Fragmentation Assay Kit (Clontech, Mountain View, CA) was used according to the manufacturer’s instructions. The fragmented, fluorescein-labeled DNA was visualized with an alkaline phosphatase-conjugated sheep anti-FITC antibody (1:1,000 dissolved in 10% fetal calf serum/PBS; Roche). As a positive control, sections were treated with DNase I (300 U/ml; Roche) for 10 min at room temperature.

### Quantification

All measurements, including Calbindin fluorescence intensities, real and outer cerebellar circumference, EGL thickness, and number of BrdU positive cells were performed with the analySIS Five software from Soft Imaging System. Embryos injected and electroporated with dsRNA derived from *Endoglycan* were compared with control-treated embryos, injected and electroporated with the EGFP plasmid only, and untreated controls. For statistical analyses, one-way ANOVA with Tukey’s multiple comparisons test (GraphPad software) was used. Values are given as mean ± SEM. 1 asterisk: *P* < 0.05. 2 asterisks: *P* < 0.01. 3 asterisks: *P* < 0.001.

## Results

### *Endoglycan* Is Expressed Widely in the Developing Cerebellum

**FIGURE 1:**
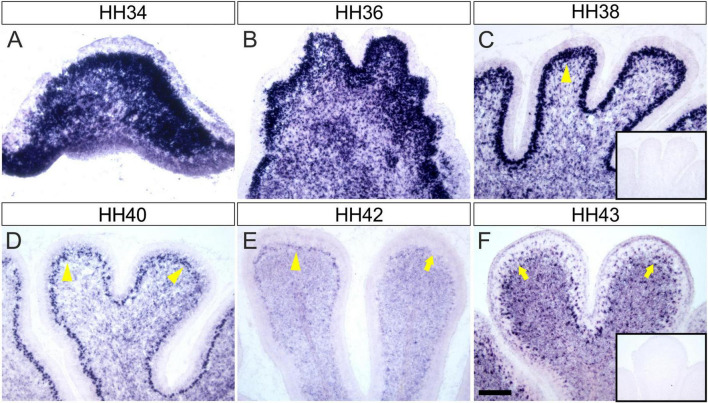
Endoglycan is expressed in different cell types during cerebellar development. **(A)** Endoglycan mRNA was detected already at early stages of cerebellum development, when cells born in the ventricular zone start migrating radially toward the periphery of the cerebellar anlage at HH34/E8 (Hamburger and Hamilton stage 34; Hamburger and Hamilton, 1951). Endoglycan is found scattered throughout the cerebellar tissue at HH36/E10 **(B)** and HH38/E12 **(C)**. At both stages, Endoglycan is expressed also in Purkinje cells. The Purkinje cell layer is labeled with yellow arrowheads. By HH40/E14 **(D)**, Purkinje cells have completed their migration and are about to align to a single cell layer. The single cell layer is perfectly formed at HH42/E16 **(E)**. At this stage, the intensity of the Endoglycan mRNA signal is strongly reduced in some lobes (arrow). By HH43/E17 **(F)**, Purkinje cells appear to express little, if any *Endoglycan* mRNA. However, as at earlier stages, Endoglycan is still found in the inner granule cell layer and now also in interneurons of the molecular layer. Inserts show adjacent sections hybridized with the sense probe as negative control. Bar, 200 μm.

The analysis of *Endoglycan* expression in the developing chicken cerebellum revealed *Endoglycan* mRNA distribution in a dynamic pattern (Figure 1). At early stages (HH34; Figure 1A), *Endoglycan* mRNA was found throughout the cerebellar anlage. *Endoglycan* expression was maintained in migrating Purkinje cells until they reached their final destination in the periphery of the cerebellar folds (HH42; Figure 1E). Expression in granule cells was restricted to the inner granular cell layer and was maintained also at the latest stages before hatching that we tested (HH43; Figure 1F). In addition to granule cells, also interneurons in the molecular layer express *Endoglycan* mRNA until late stages.

**FIGURE 2:**
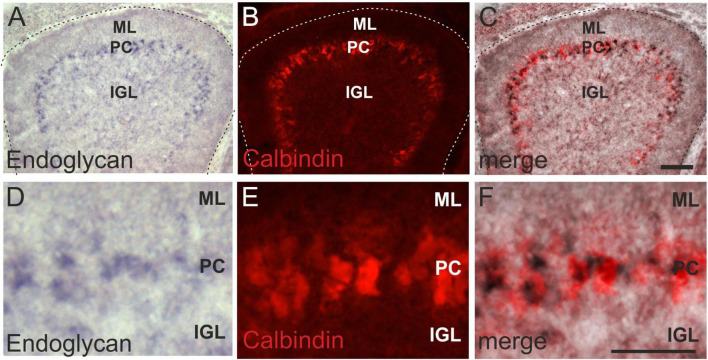
Endoglycan is expressed in Purkinje cells. The distribution of Endoglycan mRNA during cerebellar development clearly suggested expression in Purkinje cells **(A)**. However, we verified this by combining the *in situ* hybridization with Calbindin staining, a marker for Purkinje cells **(B)**. Merged images shown in **(C)**. Higher magnification clearly reveals the overlap between *in situ* hybridization signal and Calbindin staining **(D–F)**. ML, molecular layer; PC, Purkinje cell layer; IGL, Inner granule cell layer. Bar, 100 μm in **(A–C)**, 50 μm in **(D–F)**.

Expression in Purkinje cells was confirmed by co-localization of the *in situ* signal for *Endoglycan* mRNA with the Purkinje cell marker Calbindin (Figures 2A–F). At HH38, Purkinje cells have migrated to the periphery of the folds but are not yet aligned to a single cell layer (Figures 2D–F).

### *Ex ovo* RNAi Allows for Specific Knockdown of Endoglycan in the Developing Cerebellum

In ovo RNAi is an efficient method developed for silencing of candidate genes in the developing spinal cord (Pekarik et al., 2003; Wilson and Stoeckli, 2011; Andermatt et al., 2014). However, because the cerebellum starts developing in the chicken embryo only after 1 week (HH34, E8), it is not the method of choice to knockdown candidates genes in the developing brain. The embryo has already grown considerably and the head is not readily accessible through the window in the eggshell. For this reason, we developed *ex ovo* RNAi. In order to have access to the embryo for manipulations of the brain, the embryo is transferred with egg yolk and albumen to a domed plastic dish (Baeriswyl and Stoeckli, 2008). We have successfully used this method before to demonstrate a role of Contactin-2/Axonin-1 in parallel fiber development (Baeriswyl and Stoeckli, 2008).

### Endoglycan Is Required for Purkinje Cell Migration

**FIGURE 3:**
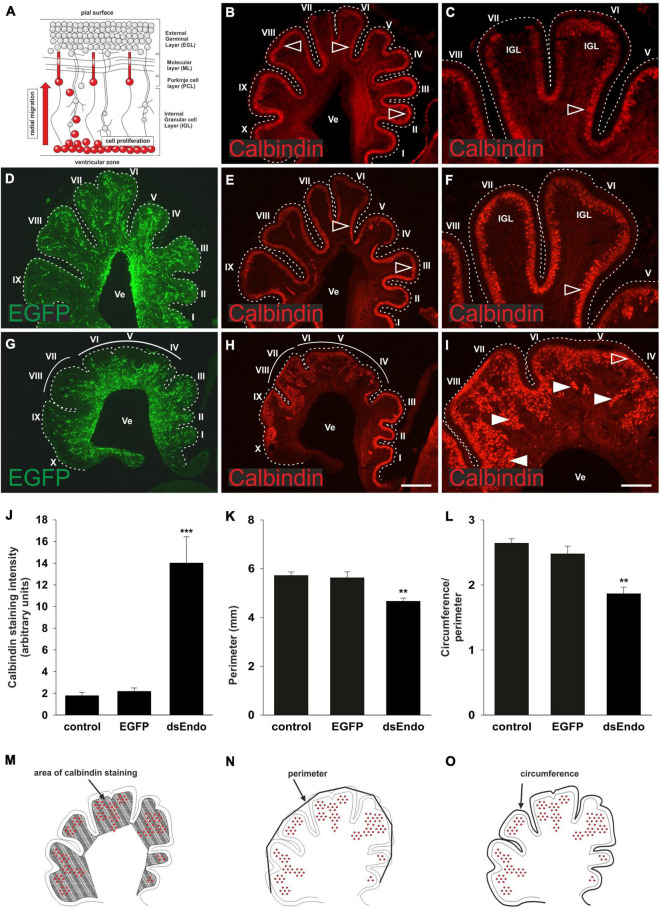
Endoglycan is required for Purkinje cell migration. Purkinje cells are born in the ventricular zone and migrate radially toward the periphery of the cerebellum, while granule cells proliferate in the external germinal layer (EGL; **A**). In the cerebellum of untreated chicken embryos sacrificed at HH38, Purkinje cells have reached the periphery of the developing folds **(B)**. Higher magnification of lobes VI and VII reveals that cells have reached their destination but have not yet finished the alignment to a single cell layer **(C)**. The developmental trajectory and the distribution of Purkinje cells is not different in control-treated embryos sacrificed at HH38. Control-treated embryos were injected and electroporated *ex ovo* at HH34 with the EGFP-expressing plasmid alone **(D–F)**. In embryos injected and electroporated with the EGFP plasmid and dsRNA derived from Endoglycan (dsEndo), the cerebellum failed to develop the characteristic lobes. They were much shorter and failed to segregate properly **(G–I)**. There was a striking failure of Purkinje cells to migrate toward the periphery. Arrowheads indicated clusters of Purkinje cells that failed to migrate and reach the periphery of the cerebellum. Open arrowheads indicated the correct location of Purkinje cells. For quantification, we measured the Calbindin-positive areas as a proportion of the area of the lobes in parasagittal sections **(J,M)**. There was no difference between the distribution of Purkinje cells, measured as Calbindin-positive pixels between non-treated and control-treated embryos (1.88 and 2.29, respectively). However, the area of the folds containing Purkinje cells was drastically increased, despite the fact that the area of the lobes was strongly reduced (14.05; ***p = 0.0008 for dsEndo vs. EGFP, ***p = 0.0004 for dsEndo vs. non-treated control). The circumference of parasagittal sections of the cerebellum **(K)**, as outlined in **(N)** was significantly smaller for the sections taken from embryos injected and electroporated with dsEndoglycan (4.67 compared to 5.72 for untreated and 5.63 for EGFP controls; **p = 0.0043 for dsEndo vs. EGFP controls, **p = 0.0013 for dsEndo vs. non-treated controls). Finally, to have a relative measure for the lack of lobe separation, we divided the outer circumference (as shown in **N**) and divided the value by the circumference of the same parasagittal sections when the length of the folds was included **(L,O)**. Values were 2.64 for untreated controls, 2.48 for EGFP expressing controls, 1.87 for dsEndo group. **p = 0.0026 for dsEndo vs. EGFP, ***p = 0.0002 for dsEndo vs. non-treated controls. Lobes are labeled by Roman numbers. IGL, inner granule cell layer; Ve ventricle. One-way ANOVA with Tukey’s multiple comparisons test used for statistical analysis.

Purkinje cells are born in the ventricular zone of the cerebellar anlage (Butts et al., 2014; Fleming and Chiang, 2015; Leto et al., 2016). From there, they migrate radially toward the cerebellar surface to form the distinct Purkinje cell layer (Figure 3A). At HH 38, Purkinje cells have migrated toward the periphery of the lobes. They are not yet aligned to a single cell layer, but very few, if any, Purkinje cells were still migrating in the cerebellum of untreated control chicken embryos (Figures 3B,C). The same was true in control-treated embryos injected with the EGFP-expression plasmid only (Figures 3D–F). In contrast, large numbers of Purkinje cells were still found in the center of the lobes in sections taken from HH38 embryos treated with dsRNA derived from *Endoglycan* (Figures 3G–I). In addition, the gross morphology of the cerebellum was severely altered. The lobes failed to grow and some of them failed to separate. Overall, the size of the cerebellum was significantly reduced. To quantify the failure to migrate, we compared the Calbindin-positive area of the cerebellum between the different groups. There was no difference between the untreated and the control-treated, GFP-expressing embryos. The Calbindin-positive area of the cerebellum was about 7 times bigger in the sections taken from embryos electroporated with dsEndoglycan (Figures 3J,M). We quantified the size of the cerebellum in two different ways. When we measured the perimeter of the parasagittal sections, we found a significant reduction in the absence of Endoglycan (Figures 3K,N). And similarly, when we quantified foliation by dividing the circumference by the perimeter of the cerebellum, we again found a significant reduction in the experimental group (Figures 3L,O). Taken together, these *in vivo* results demonstrate a role of Endoglycan in Purkinje cell migration.

### Aberrant Migration of Purkinje Cells Reduces Granule Cell Proliferation

**FIGURE 4:**
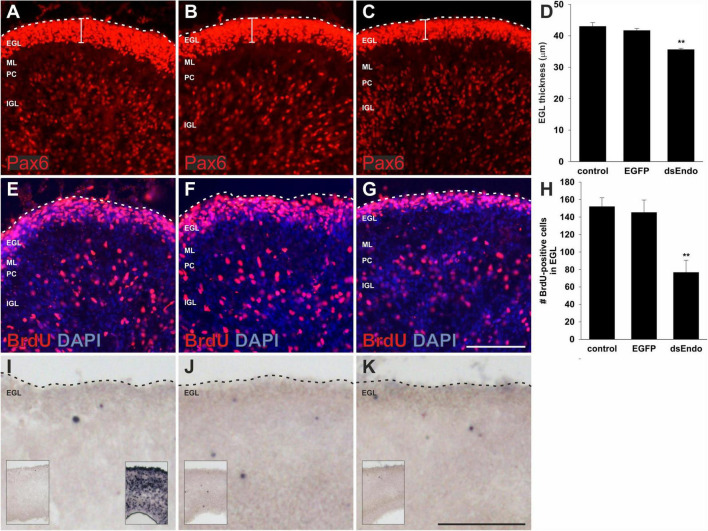
The external germinal layer is reduced due to a reduction in granule cell proliferation after silencing Endoglycan. The width of the external germinal layer (EGL) was measured in sections taken from embryos sacrificed at HH35, 1 day after *ex ovo* electroporation **(A–D)**. Pax6 stains proliferating granule cells in the EGL of untreated control embryos **(A)**, control-treated, EGFP-expressing embryos **(B)**, and in embryos injected and electroporated with dsEndoglycan (dsEndo; **C**). The thickness of the EGL was significantly reduced in embryos lacking Endoglycan **(D)**. Thickness of the EGL was determined as 43 μm in non-treated and 41.7 μm in control-treated brains, compared to only 35.6 μm width in the dsEndo group (**p = 0.0023 for dsEndo vs. non-treated, and **p = 0.0096 for dsEndo vs. EGFP-expressing controls). The decrease in granule cell proliferation was confirmed by staining for BrdU. The number of BrdU-positive cells was significantly lower in dsEndo-treated embryos (**G,H**; 76.6), compared to untreated (**E**; 152) and control-treated embryos (**F**; 145.5). **p = 0.0021 dsEndo vs. untreated, **p = 0.0085 dsEndo vs. EGFP-expressing controls. We also compared apoptosis in the cerebellum of untreated **(I)**, control-treated **(J)**, and dsEndo-treated **(K)** embryos. We found no contribution of cell death to the number of granule cells, as we did not see apoptosis at this stage of development. The inserts in **(I–K)** show lower magnification overviews. The insert in the right corner of (I) shows a positive control, where DNA fragmentation was induced with DNase treatment. One-way ANOVA with Tukey’s multiple comparisons test used for statistical analysis. EGL, external germinal layer; ML, molecular layer; PC, Purkinje cell layer; IGL, inner granule cell layer. Bar, 100 μm.

**FIGURE 5:**
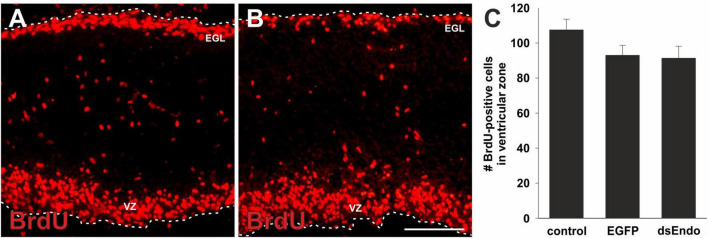
The lack of proliferation in the absence of Endoglycan is specific for granule cells. To rule out a general effect of Endoglycan on cell proliferation, we also quantified the number of BrdU-positive cells in the ventricular zone (VZ). At HH35, there are many BrdU-positive cells in the external granule cell layer (EGL), but also in the ventricular zone. We found no significant difference in the number of proliferating cells in the ventricular zone in sections taken from controls **(A)** compared to embryos lacking Endoglycan (dsEndo; **B**, *p* = 0.1978 and 0.9873). Bar: 100 μm. **(C)** We counted an average of 107.5 ± 6.1 cells in untreated embryos (*n* = 7), 93.0 ± 5.7 cells in control-treated, EGFP-expressing embryos (*n* = 3), and 91.3 ± 6.9 cells in dsEndo-treated embryos (*n* = 5). One-way ANOVA with Tukey’s multiple comparisons test used for statistical analysis.

We hypothesized that aberrant Purkinje cell migration and failure to establish the Purkinje cell layer in the periphery of the cerebellar folds prevented cerebellar growth and the formation of the lobes. Purkinje cells are suggested to regulate the proliferation of granule cells (Dahmane and Ruiz i Altaba, 1999; Wallace, 1999; Wechsler-Reya and Scott, 1999; Lewis et al., 2004). It was demonstrated that Shh (Sonic hedgehog) released by Purkinje cells affected proliferation of granule cells in the outer EGL (external granule cell layer). In turn, reduced proliferation of granule cells was shown to result in changes of cerebellar morphology similar to the ones we observed after downregulation of Endoglycan (Lewis et al., 2004; De Luca et al., 2016; Figure 3). A reduced rate of granule cell proliferation was indeed what we found in embryos after silencing *Endoglycan*. When we used Pax6 as a marker for granule cells, we found a thinner EGL in experimental embryos compared to control-treated and untreated embryos (Figures 4A–D). This decrease in EGL width was due to a reduced proliferation rate of granule cells rather than apoptosis (Figure 4). When we compared BrdU-positive cells in the EGL, we found no difference between untreated (Figure 4E) and control-treated embryos (Figure 4F). However, there were only about half as many proliferating granule cells in the EGL of embryos after electroporation of dsEndoglycan (Figures 4G,H). Apoptosis did not contribute to the decrease in granule cell number, as we did not find any cell death in the cerebellum of control or experimental embryos at these stages (Figures 4I–K). In contrast to granule cells, the proliferation rate of Purkinje cells and other cells born in the ventricular zone at HH35 did not differ between control embryos and embryos lacking Endoglycan (Figure 5). These results are consistent with a lack of Shh provided by Purkinje cells as a reason for the decrease in granule cell proliferation.

Taken together, our *in vivo* experiments demonstrate that Purkinje cell migration in the developing cerebellum requires Endoglycan. In its absence, Purkinje cells fail to reach their target positions in the periphery. The observed stunted growth of the cerebellum and the failure to develop the lobes properly could be explained due to a lack of Shh-induced proliferation of granule cell precursors.

## Discussion

In summary, our results demonstrate a vital role for Endoglycan in Purkinje cell migration in the developing cerebellum. In agreement with the results of our *in vivo* studies in the developing spinal cord (Baeriswyl et al., 2021), the observed phenotype is consistent with the hypothesis that Endoglycan is an essential regulator of cell-cell contacts by modulating the strength of adhesion between cells. This model is supported by observations *in vitro* (Baeriswyl et al., 2021). In analogy to our findings in the developing spinal cord (Baeriswyl et al., 2021) we suggest a role of Endoglycan as a regulator of adhesive strength also in the cerebellum. In the absence of Endoglycan, the adhesion between precursor cells in the cerebellar anlage is too strong for Purkinje cells to migrate properly. This failure to migrate results indirectly in a reduced proliferation rate of granule cell precursors. Because Purkinje cells get stuck in the center of the folds along their migratory path, they fail to deliver Shh to the external granule cell layer, where granule cell precursors are located during proliferation. Reduced granule cell proliferation in the absence of sufficient Shh was previously demonstrated to result in very similar cerebellar phenotypes, with merged folds and reduced size (Dahmane and Ruiz i Altaba, 1999; Wallace, 1999; Wechsler-Reya and Scott, 1999; Lewis et al., 2004; De Luca et al., 2016).

We have not analyzed whether the migration of interneuron precursors giving rise to stellate and basked cells would be perturbed as well in the absence of Endoglycan. Because these cells migrate over a longer period of time and not as a “wave,” a reduced migration rate would be very difficult to detect. Therefore, we focused on the analysis of Purkinje cells. However, we do not suggest a cell-autonomous effect of Endoglycan in Purkinje cells. Our results shown here are comparable to our previous findings in the spinal cord, where the level but not the source of Endoglycan mattered for the navigation of commissural growth cones at the ventral midline, the floor plate (Baeriswyl et al., 2021).

The fine-tuning of adhesive strength for migrating cells or extending neurites is an important aspect of neural circuit formation. Not only growth cones, but also cells need to reach distant destinations. In the cerebellum, in contrast to the cortex, cells are also born in the periphery, not only in the ventricular zone (Sotelo, 2011; Hashimoto and Hibi, 2012; Butts et al., 2014; Marzban et al., 2015; Leto et al., 2016). Granule cell precursors migrate tangentially from the rhombic lip to form the external granular layer, where they respond to Shh, a proliferative signal for granule cell precursors. Once they cease to proliferate and mature, they start to extend processes parallel to the cerebellar surface. At a slightly later stage, they form a third process, this time parallel to the Bergman glia fibers. Mature granule cells then slide along the glial fibers toward the center of the lobes by crossing the developing Purkinje cell layer to form the internal granule cell layer.

Purkinje cells have to migrate in the opposite direction during their radial migration from the ventricular zone. The appropriate localization of Purkinje cells appears to be essential for cerebellar function (Fleming and Chiang, 2015). Purkinje cells integrate the afferent information received directly from climbing fibers, or indirectly from mossy fibers, relayed by granule cells. The spontaneous mouse mutants reeler and scrambler demonstrate the importance of Purkinje cell location for cerebellar function, as the abnormal behavior of these mice due to aberrant motor control was linked to aberrant positioning of Purkinje cells (Goldowitz et al., 1997; Miyata et al., 2010). However, the cerebellum does much more than motor control and motor learning. Non-motor functions of the cerebellum, that is, its contribution to cognition and language, have been widely recognized by now (Schmahmann, 1991; Strick et al., 2009; Buckner, 2013). Therefore, it comes as no surprise that changes in cerebellar structure and function have been linked to many neurodevelopmental disorders, such as attention deficit-hyperactivity disorder (ADHD) or autism spectrum disorders (ASD) (Courchesne et al., 2005; Fatemi et al., 2012; Stoodley, 2016). Aberrant Purkinje cell localization has been identified as one of the recurrent findings in autistic patients (Wang et al., 2014; Thabault et al., 2022).

Despite of the importance of Purkinje cell migration for cerebellar development and function, its molecular basis is still poorly understood (Hatten, 1999; Sotelo, 2004; Rahimi-Balaei et al., 2018). In contrast, to granule cells, where a number of cell adhesion molecules contributing to cell migration or neurite extension have been identified, much less is known about Purkinje cells. Astrotactin was shown to slow down migration of granule cells in an N-Cadherin-dependent manner (Fishell and Hatten, 1991; Horn et al., 2018). Similarly, an effect of NB3/Cntn6 (Sakurai et al., 2009), Cadherin-2 (Rieger et al., 2009), NrCAM and NgCAM on granule cell migration was found in knockout mice (Sakurai et al., 2001), but Purkinje cell migration was not disturbed in any of these lines. Cell adhesion molecules of the Contactin family have been shown to affect parallel fiber development (Baeriswyl and Stoeckli, 2008; Stoeckli, 2010) but there are no reports on differences in Purkinje cell migration.

Aberrant positioning of Purkinje cells and similar cerebellar morphologies as the one described in this study were found in reeler and scrambler mice (Goffinet et al., 1984; Goldowitz et al., 1997; Goldowitz and Hamre, 1998). In these mice, the expression of Reelin by granule cells, or the expression and function of the receptors in Purkinje cells are perturbed and result in a failure of Purkinje cells to reach their target layer. In addition, defects in Purkinje cell migration have been shown in the absence of αN-Catenin (Park et al., 2002).

Most interesting in the context of our findings is a report by Sergaki and Ibáñez (2017) where GFRα1 was shown to affect Purkinje cell migration by counteracting NCAM. According to their observations, homophilic NCAM interactions negatively regulate migration. Migration can be enhanced by either modifying NCAM with poly-sialic acid (PSA) or by direct interaction with GFRα1. Poly-sialic acid is well known as a regulator of cell adhesion, not only for homophilic NCAM-NCAM interactions but also for heterophilic interactions of NCAM (Rutishauser et al., 1988; Brusés and Rutishauser, 2001; Burgess et al., 2008).

The expression of PSA-NCAM compared to non-PSA-NCAM is temporally regulated during development. Similarly, we found expression of Endoglycan during Purkinje cell migration, in line with an effect of Endoglycan as a negative regulator of cell-cell adhesion or “lubricant”. Interestingly, Endoglycan function could be controlled by ADAM10, which was shown to shed Endoglycan from the cell surface (Hsia et al., 2021).

Thus, it is still unknown which of the large number of cell adhesion molecules are required for the migration of Purkinje cells to their final position. However, our results are comparable to the effect of Endoglycan in commissural axon guidance, where Endoglycan was shown to negatively regulate adhesive strength (Baeriswyl et al., 2021). The same mechanism appears to allow for the migration of Purkinje cells to their final position.

## Data Availability Statement

The original contributions presented in this study are included in the article/supplementary material, further inquiries can be directed to the corresponding author.

## Ethics Statement

The animal study was reviewed and approved by the Cantonal Veterinary Office, Canton of Zürich.

## Author Contributions

TB, MS, and SL carried out experiments. AD and ES wrote the manuscript. All authors contributed to the article and approved the submitted version.

## Conflict of Interest

The authors declare that the research was conducted in the absence of any commercial or financial relationships that could be construed as a potential conflict of interest.

## Publisher’s Note

All claims expressed in this article are solely those of the authors and do not necessarily represent those of their affiliated organizations, or those of the publisher, the editors and the reviewers. Any product that may be evaluated in this article, or claim that may be made by its manufacturer, is not guaranteed or endorsed by the publisher.
